# Tensegrity Modelling and the High Toughness of Spider Dragline Silk

**DOI:** 10.3390/nano10081510

**Published:** 2020-07-31

**Authors:** Fernando Fraternali, Nicola Stehling, Ada Amendola, Bryan Andres Tiban Anrango, Chris Holland, Cornelia Rodenburg

**Affiliations:** 1 Department of Civil Engineering, University of Salerno, 84084 Fisciano (SA), Italy; adamendola1@unisa.it; 2 Department of Materials Science & Engineering, University of Sheffield, Sir Robert Hadfield Building, Mappin Street, Sheffield S1 3JD, UK; nastehling1@sheffield.ac.uk (N.S.); christopher.holland@sheffield.ac.uk (C.H.); c.rodenburg@sheffield.ac.uk (C.R.); 3 Centre for Biomedical and Chemical Science School of Science, Auckland University of Technology, Auckland 1010, New Zealand; andres.tiban.anrango@aut.ac.nz

**Keywords:** spider silk, scanning electron microscopy, plasma etching, mesoscale modelling, tensegrity systems, biomimetic fibres

## Abstract

This work establishes a tensegrity model of spider dragline silk. Tensegrity systems are ubiquitous in nature, being able to capture the mechanics of biological shapes through simple and effective modes of deformation via extension and contraction. Guided by quantitative microstructural characterization via air plasma etching and low voltage scanning electron microscopy, we report that this model is able to capture experimentally observed phenomena such as the Poisson effect, tensile stress-strain response, and fibre toughness. This is achieved by accounting for spider silks’ hierarchical organization into microfibrils with radially variable properties. Each fibril is described as a chain of polypeptide tensegrity units formed by crystalline granules operating under compression, which are connected to each other by amorphous links acting under tension. Our results demonstrate, for the first time, that a radial variability in the ductility of tensegrity chains is responsible for high fibre toughness, a defining and desirable feature of spider silk. Based on this model, a discussion about the use of graded tensegrity structures for the optimal design of next-generation biomimetic fibres is presented.

## 1. Introduction

It is generally accepted that the remarkable mechanical performance of spider silk dragline originates from a hierarchical organization of proteins into a hydrogen bonded structure of ordered crystalline β-sheets, embedded in a disordered amorphous matrix [1,2,3,4,5,6]. However, at the mesoscale, it has also been shown that silk assembles into nanofibrils with diameters ranging from ~30 nm [3] to more than 100 nm [2,5] and that a fibre has structurally and functionally distinct regions; a load bearing core (consisting of inner (1800–2300 nm), and outer (300–400 nm) sections [2,7,8]) surrounded by protective lipid (10–20 nm), glycol (40–100 nm), and skin (50–100 nm) layers [2]. 

From a biomimetic perspective, the mechanical properties of spider silk are often held as a gold standard for industrial fibre production (see the recent review paper [9]). The review studies presented in [10,11] report values of spider dragline silk tensile strength and toughness that range in the intervals 1.1–1.8 GPa and 100–400 MJ/m^3^, respectively (Table 1). In dry conditions, spider silk dragline is instead generally assumed to conserve its volume under stretching, which implies the presence of a Poisson’s effect associated with the lateral contraction of the fibre [12,13]. An investigation into the elastic response of different types of spider silk has shown that the Poisson’s ratio of the cross-section area during stretching can vary from 0 up to 1.52 [14]. This suggests that the Poisson’s ratios of silk fibres may range well outside the interval [−1.0, 0.5], making them hyperelastic isotropic materials [15]. This is most likely a consequence of the composite nature of the fibre, which induces a strongly anisotropic response of the material during deformation [16]. Of particular interest though is the link between the Poisson’s ratio of the whole silk fibre and that of the individual fibrils, since it has been reported in literature that the latter maybe significantly greater than the former [5]. Uncovering this relationship is important, as unusual, positive, or negative Poisson’s ratios are actively being used to design mechanical metamaterials exhibiting unconventional mechanical behaviours (refer, e.g., to [17] and references therein).

Whilst there have been several concerted modelling efforts to relate silk’s structures to its mechanical properties [3,4,5,6,7,8,18,19], accounting for the precise contributions of each of these structural elements on a fibre’s mechanical response has been inconclusive. Consider, e.g., the role played by the interfaces between the fibrils, which some studies define as mechanically weak and responsible for easy slippage of adjacent fibrils [6], while other studies observe the presence of heterogeneous protrusions along such surfaces determining a non-slip kinematics and energy dissipation due to interlocking effects [20]. In addition, while the literature to-date is populated by a number of multiscale and hierarchical approaches to the mechanics of the dragline silk [1,2,3,4,5,6,18], and there is experimental evidence of the partitioning of the spider silk fibres into layers with different mechanical behaviours [7,8], one observes that the grading of the mechanical properties along the radial coordinate of silk fibres has not been extensively investigated. 

Experimentally, a granular-type, hierarchical model of spider dragline has been proposed in [5], on the basis of microstructural atomic force microscopy (AFM) and scanning electron microscopy (SEM) characterization [7]. Such a model effectively explains the formation of spider silk fibrils in the form of chains of crystalline granules, whose geometry appreciably changes during the synthesis of the material as it moves through the gland, silk duct, and finally as the fibre. However, the model presented in [5] does not attempt to characterize the experimentally observed anisotropic response of the material [14].

Insights into these critical challenges may be provided through the window of tensegrity modelling. Most of the available mechanical models of silk adopt either particle-type models and coarse-graining approaches, or finite element models (see, e.g., the review paper [18]). The tensegrity paradigm leads to an alternative type of modelling, which describes a spider fi as a 3D continuum composed of lower-order continua. The latter consist of 1D rods carrying tensile and compressive forces, hereafter respectively named tendons and struts. Skelton and Nagase [19] have observed that a tensegrity model of a spider silk fibre that accounts for the transverse stiffening effect played by the crystalline granules leads to an increase of the overall tensile stiffness, as compared to a tendon-only model. 

The present work introduces, for the first time, graded tensegrity modelling of the spider dragline fibre informed by the results of plasma etching and low-voltage SEM microstructure identification tests [7]. The proposed model describes the generic silk fibril as a chain of tensegrity units composed of axial and oblique tendons, and transverse struts. The size of the tensegrity granules varies along the radial coordinate, and matches the nanostructures revealed by SEM imaging after exposure of the fibre to plasma etching, which have shown to be related to fibre mechanical properties [7]. 

Our proposed tensegrity model not only replicates Poisson’s effect under longitudinal stretching [12,13,14], but also accurately reproduces the experimental stress vs. strain response of the fibre under tensile loading and captures the enhanced toughness of the material through crack-deflection [6] and crack-stopper [21] mechanisms. The given model relates such mechanisms to regional-dependent deformation modes of the fibrils, and scale effects for the fibrils’ ductility [22,23]. In particular, such a model explains the mismatches occurring between the Poisson’s ratios of the individual fibrils and the overall fibre [5]. It makes a first step towards the modelling of the three-dimensional anisotropic response of silk fibres [14] through simple, uniaxial (tension/compression) force mechanisms at the nanoscale [24].

## 2. Microstructural Characterization of the Nephila Dragline Silk through Plasma Etching

An earlier study on plasma etching tests (40 kHz, 100 W, and 0.3 mbar pressure) and low voltage (LV) SEM Secondary Electron Hyperspectral Imaging (SEHI) of silk fibres spun by a single mature *Nephila inaurata* female has been presented in Ref. [7] revealing important links between ordered nanostructures and mechanical properties in fibres reeled under different conditions. Here, a reduction of the power to 66 W permitted gradual plasma etching and imaging to be performed on the same fibre, revealing nanostructural changes within the fibre cross-section.

**Figure 1:**
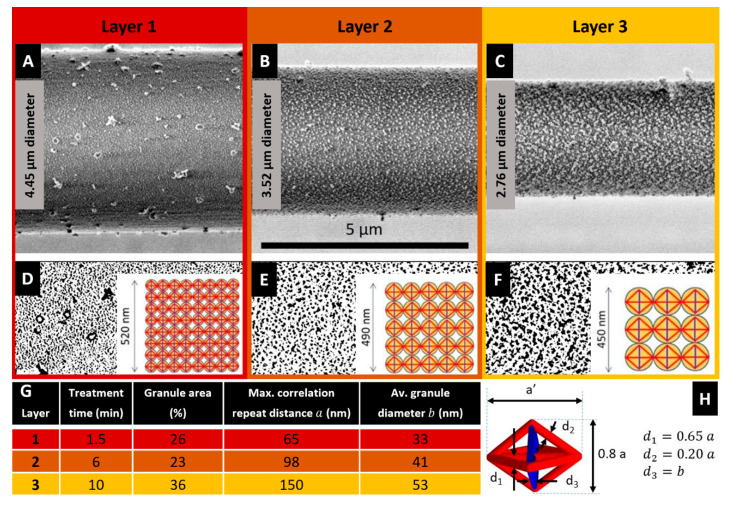
(**A**–**C**) SEM micrographs obtained after plasma etching at different exposure times of the same Nephila inaurata silk fibre. The analysed fibre had an initial diameter of 5.02 μm and was spun at a reeling speed of 20 mm/s. (**D**–**F**) Particle analysis of the nanostructures observed in the above panels respectively. Insets: Variable size of the proposed tensegrity units. (**G**) Statistical analysis of size and distribution of the crystalline granules nanostructures the proposed tensegrity units are based upon. (**H**) Geometry of a generic unit: a and a’ denote the transverse and longitudinal dimensions of the unit, respectively; d_1_, d_2_ and d_3_ indicate the diameters of the individual members.

Figure 1A–C illustrates LV-SEM micrographs (see experimental details in Supporting Information (SI), p.1) of the lateral surface of the plasma etched fibre at different etching times and the current diameter of the fibre, while Figure 1B–F shows the particle analysis of the nanostructures observed in the etched fibre. One recognizes the white regions in the Figure 1A–C, corresponding to granules consisting of ordered microcrystalline domains assumed to contain both, β-sheet structures and glycine residues [25] forming a microcrystalline area also described as a nonperiodic lattice (npl) crystallite structure [26] or a collection of small staggered sections of β-sheet [27]. These crystallite domain structures are seen to align in chains to form nanofibrils; see SI, Figure S1. For quantitative image analysis, the images are converted into binary images in which the black regions represent the granules of microcrystalline domains (Figure 1D–F).

According to the studies on the multilayer organization of the spider dragline of *Nephila clavipes* [2,7], the image shown in Figure 1A belongs to the core region of the fibre, with the layer visible after 1.5 min treatment (layer #1) most probably representing the structure present in the outer core. The layers etched for 6 min (Figure 1B) and 10 min (Figure 1C) exemplify the inner core (layers #2 and #3, respectively, the selection of representative etching times and images is described in the page 2 of supplementary information). Figure 1G shows an analysis of the size and distribution of the crystalline granules observed in the microstructures of Figure 1, which has been performed through the image processing software Fiji:ImageJ (v. 1.52t, National Institutes of Health, 9000 Rockville Pike, Bethesda, Maryland 20892, USA). This data reveals that the maximum correlation repeat distance between the microcrystalline granules *a* and their average diameter *b* monotonically increase with the treatment time. The percentage area of such granules instead also markedly increases when passing from layer #1 to layer #3, and slightly decreases when passing from layer #1 to layer #2.

## 3. Tensegrity Modelling

We now formulate a mechanical model of a spider dragline silk fibre. This combines a hierarchical organization of the material with tensegrity architectures [3] (Figure 1). The applicability of tensegrity concepts to the mechanical modelling of the spider dragline silk has been recognised previously [19,24]. This applicability is based on the observation that as silk is formed under tension by a wet spinning process and in the web the fibre dries in a stressed state, it can be assumed that the molecules are effectively locked into an oriented state by their intermolecular hydrogen bonds [28]. In the present study, we model the composite structure forming the core of the fibre as a collection of fibrils running parallel to the fibre axis [2,19], which are described as chains of tensegrity units. Given that the outer lipid, glycol, and skin layers contribute minimally to the overall mechanical response of the spider silk fibre, they are ignored here [2,7,8].

The generic tensegrity unit is formed by longitudinal and diagonal tensile elements hereafter referred to as *tendons* (or strings), marked in red in Figure 1H, and by transverse compressive struts (or bars) marked in blue. The tendons correspond to composite rods formed by amorphous chains that are attached to crystalline domains at their extremities. The struts reproduce the compressive stiffening effect that is played by β-sheet plated crystals in the circumferential direction [19]. The geometry of the units changes when moving along the radial coordinate of the fibre, in agreement with the nanostructures revealed by the SEM images illustrated in the previous section. Figure 1D–F (insets) illustrates that the tensegrity units forming layers #1, #2, and #3 are inscribed in spheres with diameters equal to 65, 98 and 150 nm based on *a* values in Figure 1G. It is worth observing that the β-sheet domains contribute significantly to both the transverse compressive stiffness and the longitudinal tensile stiffness in the fibre. Since the diameter of the longitudinal tendons range between 40–100 nm (see Figure 1) and the minimum transverse dimension of the β-crystallites is in the order of a few nm [3], the result is that at the extremities of the tendons there are clusters of ordered regions of β-sheets. Such clusters form hinged joints linking the tendons each other.

**Figure 2:**
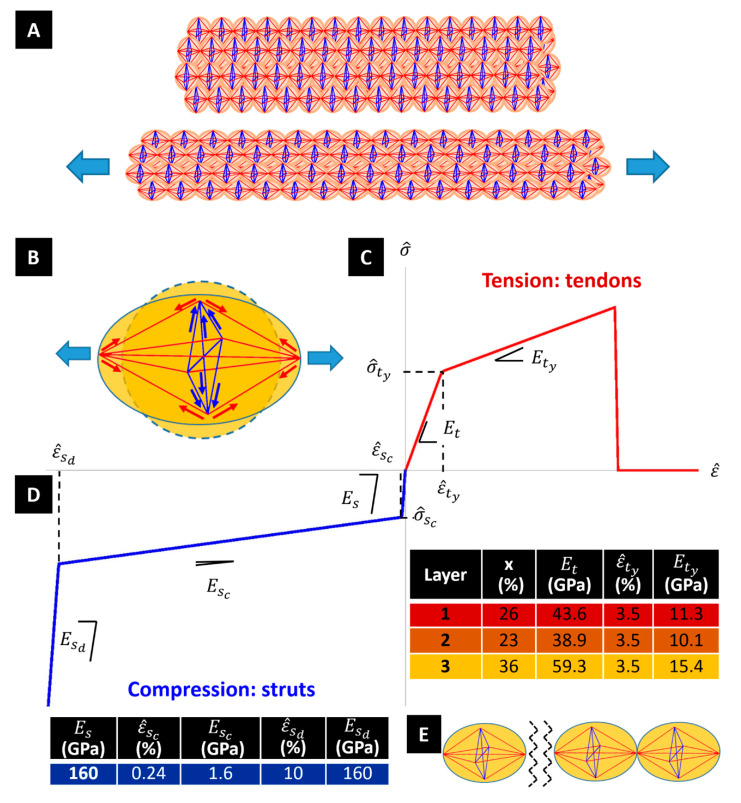
(**A**) Bundle of tensegrity fibrils (red members are tendons, while blue members are struts) and deformation mechanism under tensile loading. (**B**) The tendons are loaded in tension, while the struts are loaded in compression. Stress-strain responses of struts (**C**) and tendons **(D**) alongside their respective mechanical parameters. (**E**) Fracture of a fibril at the interface between two adjacent units.

Let us now describe the response of the fibre to a longitudinal stretching deformation through the engineering strain $\hat{\varepsilon }=\left(\ell -L\right)/L$ (positive in extension). Here, L denotes the unstretched length of the fibril, and ℓ denotes the stretched length of the fibre. Under such a loading condition, the tendons forming the tensegrity units of the fibrils are loaded in tension, while the struts are loaded in compression (Figure 2). The transverse contraction of the struts produces a radial (engineering) strain ${\hat{\varepsilon }}_{r}=\left(D-d\right)/D$, (positive in contraction) where D and d, respectively, denote the undeformed and the contracted (deformed) diameters of the fibre, respectively (Poisson’s effect) [12,13,14].

A peculiar phenomenon that affects the tensile response of dragline silk fibres is the yielding effect produced by the breaking of the (weak) hydrogen bonds forming the matrix of the fibre under stretching, which appreciably reduces the axial stiffness of the fibre [29,30]. To explain these phenomena, the current model makes use of the stress-strain responses of tendons and struts that are illustrated in Figure 2. Let us denote the elastic modulus of β-sheet crystal domains by $E_{\beta }$, and the pre-yielding elastic modulus of the noncrystalline domains by $E_{\alpha }$.

The stress-strain response of the struts is characterised by an elastic phase with Young modulus $E_{s}=E_{\beta }$, since these members are supposed to be fully crystalline. The initial elastic response extends up to a buckling event occurring when the local engineering strain reaches the critical value ${\hat{\varepsilon }}_{s_{c}}$. The buckling phase is assumed to be elastic, and is associated with a marked decrease in the axial stiffness of the member, as compared to the initial elastic phase (refer, e.g., to [31]). The post-buckling phase features an initial “soft” branch, with tangent elastic modulus $E_{s_{c}}\ll E_{s}$, and next a “hard” branch with tangent elastic modulus $E_{s_{d}}=E_{s}$. The latter describes the combined effects of the densification of the material under moderately large axial strains, and the progressive stiffening of the members in the post-buckling regime [31]. We assume that such a phase initiates when the strut’s strain reaches a given threshold value ${\hat{\varepsilon }}_{s_{d}}=10\%$ (Figure 2D). For what concerns the tendons, we characterise the initial (pre-yielding) response of such members, which are formed by a blend of crystalline and noncrystalline domains, through the following composite Young modulus, $E_{t}$:

(1) $$E_{t}=x_{\;}E_{\beta }+\left(1-x\right)E_{\alpha }$$

Here, x is the volume fraction of β-sheet crystals. The yielding of the tendons is assumed to occur when the local engineering strain reaches a threshold value ${\hat{\varepsilon }}_{t_{y}}.$ The incremental response the post-yielding regime, up to tensile failure, is described by a reduced tangent elastic modulus $E_{t_{y}}$ < $E_{t}$ [32] (Figure 2C).

On assuming $E_{\beta }=160\;\mathrm{GPa}$ [30,33], and $E_{\alpha }=2.7\;\mathrm{GPa}$ [34], hereafter we make use of the material properties listed in Figure 2D. The assumed value of $E_{\beta }$, which whilst significantly higher than the experimental Young’s moduli of β-sheet nanocrystals reported in the literature [33,35], is imported from the theoretical study presented in [30]. It refers to the rigid response of the β-crystalline domains in the pre-yield and pre-buckling regimes in tension and compression, respectively. It is worth observing that the fraction of crystalline domains reported in Figure 1 matches the percentages the crystalline granules observed in the microstructures of Figure 2.

**Figure 3:**
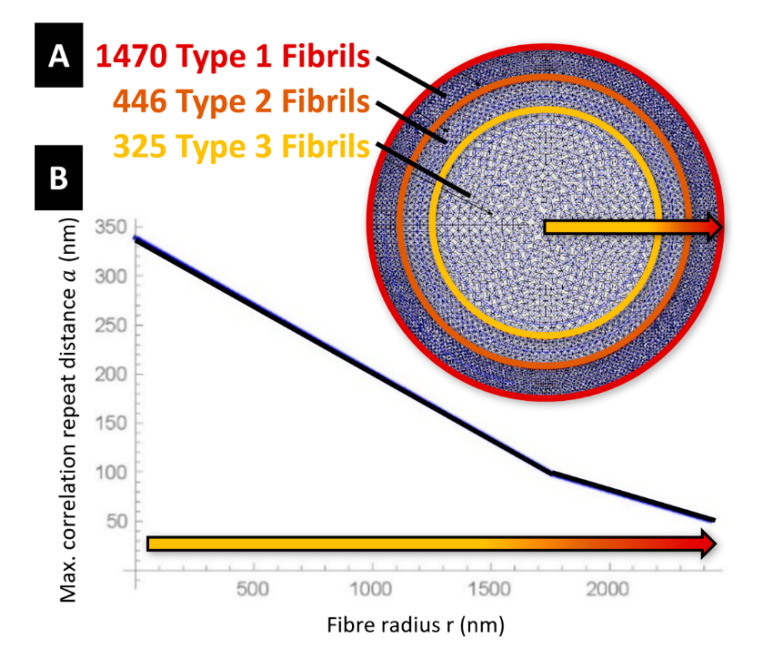
(**A**) Spatial distribution of the fibrils over the fibre cross-section. (**B**) Interpolation function of the microstructure parameter $a^{\prime }$.

The tendons and the struts are assumed to have a cylindrical shape. The diameters of such members change with the microstructure parameters a and b, as shown in Figure 1H. In particular, the diameter d of the struts is let to coincide with the average diameter b. of the microcrystalline granules. Let us now denote fibrils of Type 1, 2, and 3 the fibrils that exhibit (a=65 nm, b=33 nm), (a=98 nm, b=41 nm) and (a=150 nm, b=53 nm), respectively. The fibril distribution depicted in Figure 3 assumes that the cross-section of dragline silk fibre is composed of a packing of 1470 fibrils of Type 1 in the annulus bounded by the circle with radius 2422 nm and the circle with radius 1967 nm (layer 1: outer core of the fibre); a packing of 446 fibrils of Type 2 in the annulus bounded by the circles with radii 1967 nm and 1570 nm (layer 2: intermediate core); and a packing of 325 fibrils of Type 3 in the remaining inner core of the fibre (Figure 3A). All such packings are polar-symmetric.

The microstructure parameter $a^{\prime }$ defines the longitudinal spacing of the tensegrity units in the generic fibril, as shown in Figure 1G. We model the variation of this parameter with the radial coordinate by interpolating the values taken in correspondence with the layers #1,2,3, where we assume $a^{\prime }=a$. The interpolating function plotted in Figure 3B shows $a^{\prime }$ on the vertical axis and the radial coordinate r with origin at the centre of the fibre on the horizontal axis. One observes that it results in $a^{\prime }=339\;\mathrm{nm}$ at the centre of the fibre (r=0), and $a^{\prime }=51\;\mathrm{nm}$ at the boundary of the fibre’s core (r=2422 nm). The assumption of a piecewise constant distribution of a with the radial coordinate allows us to simplify the packing of the fibrils across the fibre; while the assumed piecewise linear variation of $a^{\prime }$ with r leads us to capture the crack deflection effect between adjacent grains [6]. We also neglect the load carrying capacity of the matrix in between the fibrils material [6,36].

It is known that the ductility of a material, as well as other key mechanical parameters, increases when the scale of the microstructure gets finer [22,23]. The SEM characterization presented in Figure 1 shows that the microstructure parameters a and b appreciably increase moving towards the centre of the fibre. This implies the presence of larger crystalline domains at the inner core than at the outer core of the fibre (see also Figure 1 and Figure 3B) [7,37,38]. We hereafter introduce a scale effect on the amplitude of the failure strain (or strain at break) of the fibrils ${\hat{\varepsilon }}_{f_{u}}$. The ductility of the material is measured by the ratio between ${\hat{\varepsilon }}_{f_{u}}$ and the yield strain of the fibril ${\hat{\varepsilon }}_{f_{y}}$. The strain parameters ${\hat{\varepsilon }}_{f_{y}}$ and ${\hat{\varepsilon }}_{f_{u}}$ refer to the tensile response of the overall fibril, and mark the first bending point of the stress-stain response, and the break strain of the fibril, respectively. Due to the geometry of the tensegrity unit shown in Figure 1H, it is clear that ${\hat{\varepsilon }}_{f_{y}}$ coincides with the yield strain ${\hat{\varepsilon }}_{t_{y}}$ of the tendons aligned with the fibrils axis, while ${\hat{\varepsilon }}_{f_{u}}$ coincides with the strain that determines the rupture of the fibril at the interface between adjacent units (Figure 2E). Characteristic values of ${\hat{\varepsilon }}_{f_{u}}$ are found in the interval 0.20–0.30 for dry spider silk fibres tested in air in the literature [10,11,39]. These estimates account for marked interspecific and intraspecific variabilities [40], which are also related to mechanical alignment parameters [29], the reeling speed [7] and testing procedures [13]. With reference to the *Nephila inaurata* silk fibres analysed in the present work, we assume that ${\hat{\varepsilon }}_{f_{u}}$ varies linearly with the radial coordinate r in the interval r∈[0, 750] nm Here, it results in (a=150 nm, b=53 nm), and the microstructure parameter a’ varies in between 339 nm (r=0) and 236 nm (r=750 nm). Specifically, we assume ${\hat{\varepsilon }}_{f_{u}}=0.220$ at r=0, and ${\hat{\varepsilon }}_{f_{u}}=0.265$ at r=750 nm. We keep ${\hat{\varepsilon }}_{f_{u}}$ constantly equal to 0.265 for r>750 nm.

## 4. Predicting Tensile Stress-Strain Response

**Figure 4:**
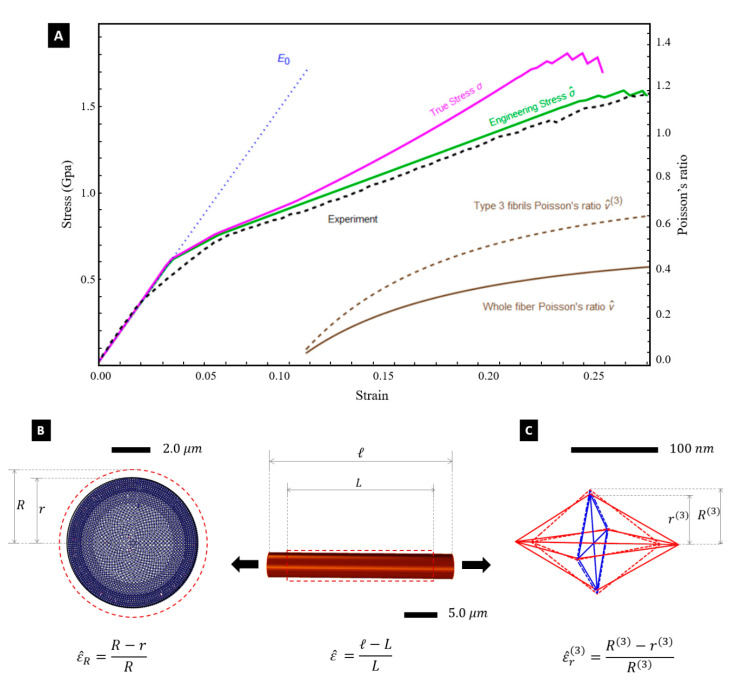
(**A**) Engineering ($\hat{\sigma }-\hat{\varepsilon }$) and true (σ−ε) stress-strain curves predicted by the tensegrity model, in comparison with the experimental results (black dashed line) for the engineering stress-strain response (exp. curve) [7]. Fluctuations of the predicted stress-strain curves at high strains (red and blue solid lines) are due to progressive fibril rupture. Secondary axis denotes the Poisson’s ratios of the whole fibre ($\hat{\nu }$) and Type-3 fibrils (${\hat{\nu }}^{\left(3\right)}$). (**B**,**C**) Different views of the deformed configurations of the whole fibre (**B**), and a Type-3 fibril (**C**) at $\hat{\varepsilon }=0.265$.

Let us employ the tensegrity model illustrated in the previous section to predict the response of the Nephila silk fibres under stretching to large deformations. We make use of the path-following algorithm in strain control presented in [41], which numerically approaches quasi-static deformations of tensegrity structures in the large deformation regime. Our predictions of the engineering and true stress-strain curves of the fibre are illustrated in Figure 4A. The engineering stress $\hat{\sigma }$ is obtained by dividing the summation of the pulling forces acting on the individual fibrils by the initial cross section area of the fibre $\hat{A}$. The true stress σ is instead obtained by dividing the total pulling force by the deformed cross section area A. Finally, the true strain ε is linked to $\hat{\varepsilon }$ through the equation: $\varepsilon =\log\left(1+\hat{\varepsilon }\right)$ [32].

**Figure 5:**
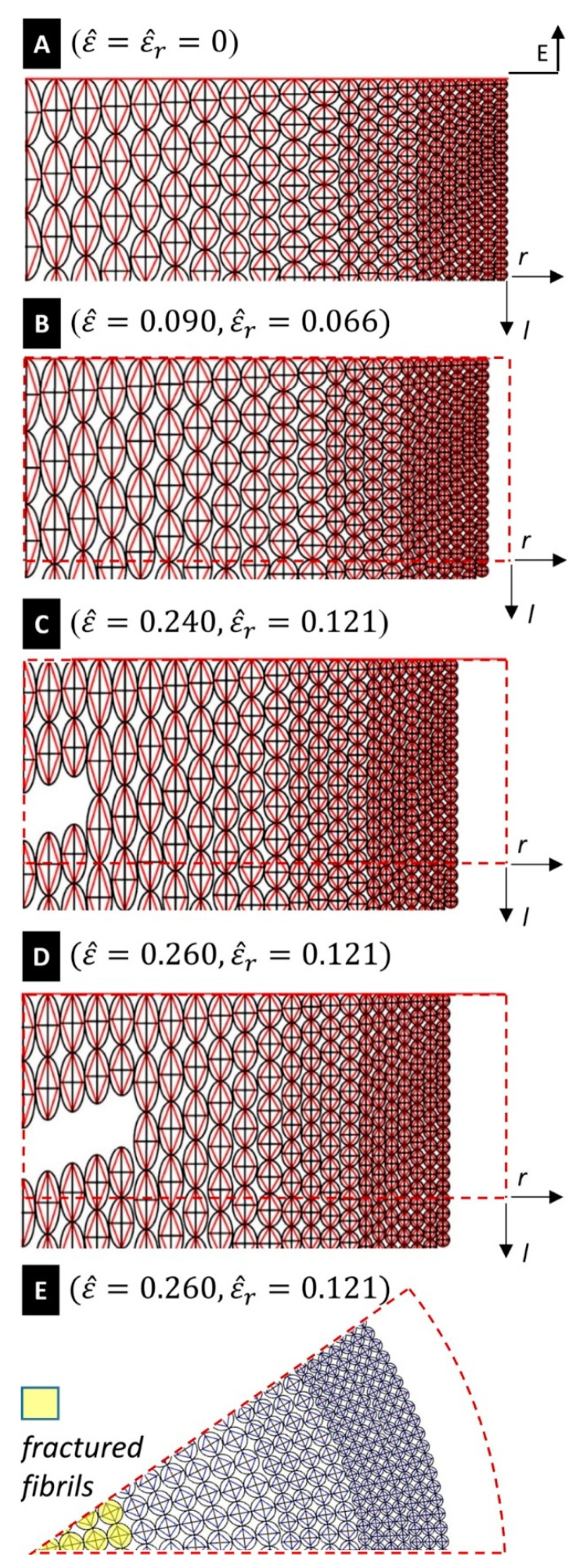
Snapshots of fibre deformation under different values of the applied longitudinal strain $\hat{\varepsilon }$. The quantity ${\hat{\varepsilon }}_{r}$ denotes the radial strain that accompanies $\hat{\varepsilon }$ due to Poisson’s effect. (**A**–**D**): Longitudinal sections of the stretched fibre in correspondence with different levels of strain. (**E**) transverse cross-section E-E of the fibre under $\hat{\varepsilon }=0.260$ and ${\hat{\varepsilon }}_{r}=0.121$ (r and l, respectively, denote radial and longitudinal axes; dashed lines mark the undeformed configuration of the fibre).

Figure 4A shows that the engineering stress-strain curve $\hat{\sigma }-\hat{\varepsilon }$ predicted by the tensegrity model is in good agreement with the experimental curve obtained through the experimental setup illustrated in [7]. Such a setup consists of a testing machine equipped with a 5 N loading cell, which stretches a fibre sample with 5 mm gauge length at the controlled strain rate of $3.3\times 10^{-2}{\;s}^{-1}$. The predicted yield strain of the fibre coincides with that of the axial tendons, i.e., it results ${\hat{\varepsilon }}_{f_{y}}={\hat{\varepsilon }}_{t_{y}}=3.5\%$. The experimentally observed value of the fibre breaking strain is 0.265, and this justifies the assumptions made about the distribution of ${\hat{\varepsilon }}_{f_{u}}$ across the core of the fibre at the end of the previous section. Selected snapshots of the deformation of the silk fibre predicted by the tensegrity model are given in Figure 5, for different values of the engineering strain $\hat{\varepsilon }$. A finer matching between the experimental and predicted yield points of the stress-strain curves in Figure 4A can be obtained by suitably grading the value of the yield strain of the tendons across the radial coordinate. We do not account for such a refinement in the present work in order to keep the tensegrity model as simple as possible.

Figure 4A also shows the predicted variation of the Poisson’s ratio $\hat{\nu }=\frac{{\hat{\varepsilon }}_{r}}{\hat{\varepsilon }}=\frac{\left(D-d\right)}{\left(\ell -L\right)}\times \frac{L}{D}$ with the engineering strain $\hat{\varepsilon }$. Here, we assume D=2×2422=4844 nm, which corresponds to the initial (undeformed) diameter of the core of the fibre (see Section 4). The deformed diameter d was computed by summing up the deformed diameters $a_{i}$ of the individual fibrils (Figure 3A). An $\hat{\nu }$ vs. $\hat{\varepsilon }$ plot is experimentally hard to obtain, resulting in experimental fluctuations [12,42], but Figure 4A highlights that the tensegrity model is actually able to predict a deformation-dependent behaviour of the Poisson’s ratio. Such a behaviour was to be expected since we are accounting for large strains [43]. One observes that $\hat{\nu }$ increases under growing axial strains, by approaching the value $\hat{\nu }$ = 0.426 at $\hat{\varepsilon }=0.265$. In parallel, we observe small and oscillating (positive/negative) values of the volumetric strain $e=\frac{V-V_{0}}{V_{0}}$ ($V_{0}$ denoting the initial volume, and V denoting the strained volume), i.e.,: e= +0.005 for $\hat{\varepsilon }=0.006,$ and e=−0.006 for $\hat{\varepsilon }=0.265$. The current model predicts nonuniform radial strains, the different fibrils that form the fibre. The largest radial strains are observed in the Type-3 fibrils occupying the inner core of the fibre (Figure 3A), which grow up to ${\hat{\varepsilon }}_{r}^{\left(3\right)}=$0.172 for $\hat{\varepsilon }=0.265$. Type-1 and Type-2 fibrils instead experience very small radial strains remaining always lower than 0.5%, up to fibre rupture. The current model indeed confines the radial deformation mechanism to the inner core formed by the type 3 fibrils, whose radius is equal to 65% of the overall fibre radius (see Figure 3). The larger dimensions of the members composing the type 3 fibrils, as compared to those characterizing the struts and tendons of type 1 and type 2 fibrils (Figure 1G,H), determines indeed the achievement of more relevant axial forces in such members. This results in a Poisson’s ratio ${\hat{\nu }}^{\left(3\right)}=\frac{{\hat{\varepsilon }}_{r}^{\left(3\right)}}{\hat{\varepsilon }}$ of the Type-3 fibrils that is actually larger than the global (or average) Poissons’s ratio $\hat{\nu }$ (Figure 4B,C), since this quantity reaches the value ${\hat{\nu }}^{\left(3\right)}=0.65$ for $\hat{\varepsilon }=0.265$. ($\hat{\nu }$ = 0.426).

The deformation mode predicted by the current model qualitatively matches the experimental results presented in [5] for *Nephila clavata* fibres, which report microfibrils Poisson’s ratios greater than 0.5, against an average Poisson’s ratio lower than 0.5. It is an easy task to prove that the following geometric relation relates the strain measures $e,\;\hat{\varepsilon },\;{\hat{\varepsilon }}_{r}$: $e=\left(\frac{\pi d^{2}\ell }{4}-\frac{\pi D^{2}L}{4}\right)/\left(\frac{\pi D^{2}L}{4}\right)={\left(1-{\hat{\varepsilon }}_{r}\right)}^{2}\left(1+\hat{\varepsilon }\;\right)-1$. By solving the equation e=0 for ${\hat{\varepsilon }}_{r}$, one gets ${\hat{\varepsilon }}_{r}=$0.111 for $\hat{\varepsilon }=0.265\;(i.e.,\;{\hat{\varepsilon }}_{r}=0.418\;\hat{\varepsilon }$). The tensegrity model approximatively reproduces such a result, by returning ${\hat{\varepsilon }}_{r}=0.113$ for $\hat{\varepsilon }=0.265$, that is, ${\hat{\varepsilon }}_{r}=0.426\;\hat{\varepsilon }$. Guinea et al. [12] report a mean value of 0.993 and a standard deviation ±0.013 for the volume ratio $\frac{V}{V_{0}}$ of *Argiope trifasciata* fibres obtained through forced silking at different spinning speeds, under longitudinal strains $\hat{\varepsilon }\in \left[0,0.45\right]$. The corresponding mean volumetric strain $e=\frac{V}{V_{0}}-1\;$is equal to −0.007. The experiments conducted by such authors highlight alternance of positive and negative values of the volume ratio $\frac{V}{V_{0}}$ in proximity of the condition with zero volumetric strain. We are led to conclude that the present model accurately predicts the fibre volume conservation in the large strain regime, being also able to reproduce the small fluctuations of the volumetric strain experimentally observed in [12].

## 5. Predicting Fracture and Toughness

We have already observed that several available predictions of fibre fracture/failure in dragline silk account for the hierarchical organization of the material into microfibrils [1,2,3,4,5,6,18]. However, the link between such a phenomenon and the variation of the size and density of the crystalline domains has only been investigated qualitatively to-date [7]. Examining the fracture response of our tensegrity model, we observe that it predicts a progressive breakage of the microfibrils, starting within the inner core of the fibre. The first fibril breaks under an engineering strain $\hat{\varepsilon }=0.22$ (see the values of ${\hat{\varepsilon }}_{f_{u}}$ given at the end of Section 3). The results in Figure 5E highlight that the portion of the core of the fibre bounded by the circle with radius r=300 nm is composed of all broken (fractured) fibrils, for $\hat{\varepsilon }=0.24$. The fractured region of the fibre extends to the region bounded by the circle with radius r=600 nm at $\hat{\varepsilon }=0.260$. As we have already noted, the current model predicts complete failure of the fibre at $\hat{\varepsilon }=0.265$, which corresponds to the experimentally observed breaking strain.

The progressive fibril ruptures give rise to the fluctuations of the predicted stress-strain curves in Figure 4, which is a common feature in experimental silk tensile testing [29,44]. Studies dealing with *Argiope trifasciata* fibres report fracture surfaces that do not show voids and cracks at the skin-core interface [36,39]. The fractographic analyses presented in [36,39] observe a “globular” (or “mosaic”) structure of the cross-section of the fractured fibre. The morphology of the fracture surface is also influenced by the applied strain rate and the presence of structural defects. Yazawa et al. observe in a recent study [45] that spider dragline silk fibres of *Nephila clavata* tend to break at macroscopic structural defects at low strain rates, and at microfibrils at faster strain rates. They also observe that during stretching, the longitudinal alignment of crystalline regions is prevalent in dry conditions, while the amorphous chains tend to appreciably align with the longitudinal axis under stretching at high relative humidity (RH) conditions. The morphologies of the fracture surfaces given in [45] highlight fibre’s rupture due breaking of microfibrils under strain rates greater or equal to $3.3\times 10^{-2}s^{-1},$ at 43% RH.

The current model predicts a mosaic-like configuration of the fractured fibre for $\hat{\varepsilon }=0.260$ (Figure 5E). It reproduces a fibril-breaking fracture mode initiating at the core of the fibre and propagates outwards. It is worth observing the tendons forming the tensegrity model formulated in the present work are made of a composite material that mixes amorphous and crystalline domains (cf. Section 3). Such members tend to align with the longitudinal axis under stretching, as shown in Figure 4C. The current assumption of graded distributions of the diameters and the failure strains of the fibrils leads us to predict a ductile tensile response of the fibre, and effectively explains its enhanced toughness [7,10,11]. This is due to the progressive deflection of the crack front along the interfaces separating fibrils with different breaking strains [6,36]. The toughness predicted by the tensegrity model up to $\hat{\varepsilon }=0.265$ amounts to 268.79 MJ/m^3^, which is just slightly greater than the experimental value of 261.97 MJ/m^3^ [7]. We refer the reader to Videos S1 and S2 of Supplementary Materials for an illustration of the stretching deformation of the fibrils of Type 3, and the whole fibre, respectively, with the latter synced with the engineering stress-strain curve of Figure 4A.

## 6. Designing Biomimetic Fibres

Researchers have been trying to recreate the properties of spider silk through artificial spinning for several decades now, with mixed success, [9,46,47,48]. In the comprehensive review by Koeppel et al. [49], the authors relayed that the general consensus across the field is that the excellent properties of many artificial silk fibres are mainly derived from a stretch-induced orientation of the fibrils, the high crystalline fraction of the material, and effective fibril linking (through the use of high molecular weight proteins).

**Figure 6:**
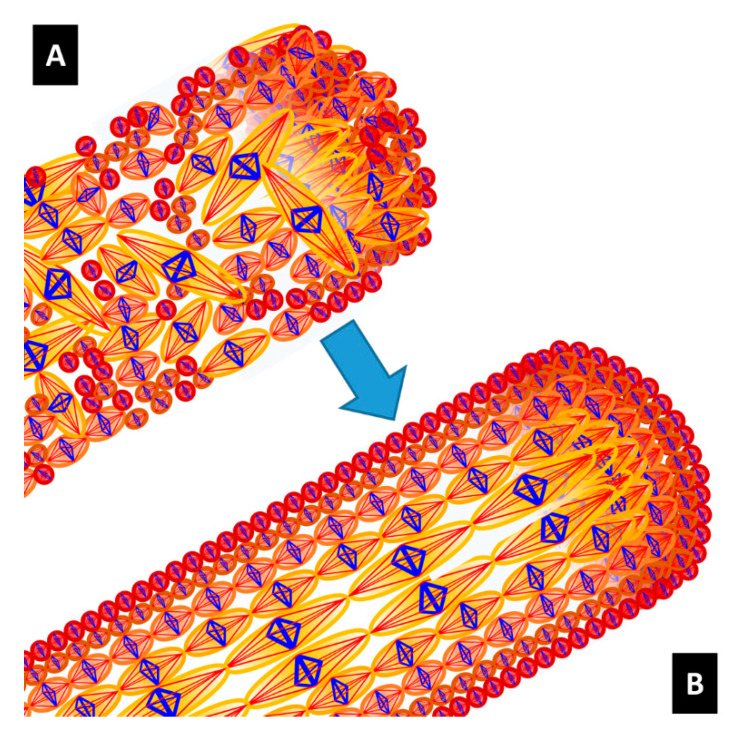
Design of artificial fibres with graded tensegrity architecture (**B**) may enable higher performance space to be accessed, as well as a graded crystalline nanostructure of the fibrils, compared to random structure (**A**).

The results of the present study highlight that it would be convenient to refine the strategy proposed in [48], with the aim of introducing a graded crystalline nanostructure of the fibrils (see Section 4). The grading of the nanostructure should be performed so as to alternate regions with large- and small-sizes of crystalline domains, by progressively reducing, e.g., the transverse dimension of the fibrils when moving towards the boundary of the fibre (see Figure 6B).

We propose that this is most readily achievable, as-in nature, through a careful control of protein interactions and applied flow fields during silk processing, essentially “locking in” structures during silk’s solidification [50,51]. Such a graded microstructure leads to a nonuniform distribution of the fibrils’ break strains, which is in turn responsible for the enhancement of the material toughness and other mechanical properties, as has been shown in Section 4.

## 7. Concluding Remarks

The present study has demonstrated that tensegrity modelling of the spider dragline silk is able to capture the experimentally observed tensile stress-strain response of the silk fibre, the Poisson effect related to the radial contraction of the cross-section, and the enhanced fracture toughness of the material. Graded modelling of spider silk ductility was able to effectively explain the enhanced toughness of the material through crack-deflection and crack-stopper mechanisms [6,20,21]. The given tensegrity model is relatively simple in terms of deformation mechanisms and the flow of forces, as it assumes the presence of only stretched and compressive members within the spider dragline fibre, and no bending stresses. It has been informed by the SEM imaging of *Nephila inaurata* silk treated with plasma etching at different times, allowing us to account for the grading of the size and the volume fraction of the crystalline domains by moving along the radial coordinate of the fibre.

Future directions of the present work will be aimed at improving the proposed model of silk fibres, which represents a first step toward a comprehensive hierarchical modelling of spider dragline silk based on tensegrity concepts. Through future work, we aim at accounting for rate- and humidity-dependent properties of struts and tendons; twisting and waving of the fibrils [3]; the inclusion of shear and bending modes [24,34], and supercontraction-induced variation of the material properties [52,53]. We also plan to model a variety of fracture initiation mechanisms, and to account for different, experimental, and theoretical predictions of the mechanical properties of the amorphous and crystalline β-sheet domains, as extensions to the present study. The enriched model is being applied to several types of natural silk, including different varieties of spider and silkworm silks. An additional extension of the present research will both outline and refine the processing parameters for artificial spinning, with the aim of developing novel biomimetic fibres with tensegrity architecture, allowing for grading of crystallinity and exceptional material toughness.

## Figures and Tables

**Table 1:** Relative density, tensile strength, and toughness of different materials.

Material	Relative Density	Tensile Strength (GPa)	Toughness (MJ/m^3^)	Toughness per Unit Mass (kJ/kg)
*Nephila inaurata* silk [7]	1.3	1.6	200–400	154–308
*Argiope trifasciata* silk [10]	1.3	1.2	100	77
*Nephila clavipes* silk [10]	1.3	1.8	130	100
Araneus silk [11]	1.3	1.1	160	123
High tensile steel [11]	7.8	1.5–3.0	6	0.77
Kevlar 49 fibre [11]	1.4	3.6	50	36

## Supplementary Materials

The following are available online at https://www.mdpi.com/2079-4991/10/8/1510/s1, Video S1: Stretching deformation of fibrils of Type 3. Video S2: Stretching deformation of the whole fibre. Document S1: Supporting Information on “Understanding low voltage SEM images” and “Optimisation of plasma exposure times”, including: Figure S1: SEM image the edge of the fibre after air plasma exposure to 66W for 1.5 min; Figure S2 left: untreated fibre, right: after 1 min exposure to plasma; Figure S3 left: after 2min exposure to plasma, right: after 6min exposure to plasma.

## References

[1] Du N., Xiang Y.L., Narayanan J., Li L., Lim M.L.M., Li D. (2006). Design of superior spider silk: From nanostructure to mechanical properties. Biophys. J..

[2] Sponner A., Vater W., Monajembashi S., Unger E., Grosse F., Weisshart K. (2007). Composition and hierarchical organisation of a spider silk. PLoS ONE.

[3] Xu G., Gong L., Yang Z., Liu X.Y. (2014). What makes spider silk fibers so strong? From molecular-crystallite network to hierarchical network structures. Soft Matter.

[4] Wang Q., Schniepp H.C. (2019). Nanofibrils as building blocks of silk fibers: Critical review of the experimental evidence. JOM-J. Min. Met. Mat. S..

[5] Lin T.Y., Masunaga H., Sato R., Malay A.D., Toyooka K., Hikima T., Numata K. (2017). Liquid crystalline granules align in a hierarchical structure to produce spider dragline microfibrils. Biomacromolecules.

[6] Fu C., Wang Y., Guan J., Chen X., Vollrath F., Shao Z. (2019). Cryogenic toughness of natural silk and a proposed structure-function relationship. Mater. Chem. Front..

[7] Stehling N., Abrams K.J., Holland C., Rodenburg C. (2019). Revealing spider silk’s 3D nanostructure through low temperature plasma etching and advanced low-voltage SEM. Front. Mater. Sci..

[8] Yazawa K., Malay A.D., Masunaga H., Numata K. (2019). Role of skin layers on mechanical properties and supercontraction of spider dragline silk fiber. Macromol. Biosci..

[9] Salehi S., Scheibel T. (2018). Biomimetic spider silk fibres: From vision to reality. Biochemist.

[10] Elices M., Pérez-Rigueiro J., Plaza G.R., Guinea G.V. (2005). Finding inspiration in Argiope trifasciata spider silk fibers. JOM.

[11] Omenetto F.G., Kaplan D.L. (2010). New opportunities for an ancient material. Science.

[12] Guinea G.V., Pérez-Rigueiro J., Plaza G.R., Elices M. (2006). Volume constancy during stretching of spider silk. Biomacromolecules.

[13] Vehoff T., Glišović A., Schollmeyer H., Zippelius A., Salditt T. (2007). Mechanical properties of spider dragline silk: Humidity, hysteresis, and relaxation. Biophys. J..

[14] Koski K.J., Akhenblit P., McKiernan K., Yarger J.L. (2013). Non-invasive determination of the complete elastic moduli of spider silks. Nat. Mater..

[15] Gurtin M.E. (1981). An Introduction to Continuum Mechanics.

[16] Cristescu N., Craciun E.M., Soós E. (2004). Mechanics of Elastic Composites.

[17] Eidini M., Paulino G.H. (2015). Unraveling metamaterial properties in zigzag-base folded sheets. Sci. Adv..

[18] López Barreiro D., Yeo J., Tarakanova A., Martin-Martinez F.J., Buehler M.J. (2019). Multiscale modeling of silk and silk-based Biomaterials-A review. Macromol. Biosci..

[19] Skelton R.E., Nagase K. (2012). Tensile tensegrity structures. Int. J. Space Struct..

[20] Cranford S.W. (2013). Increasing silk fibre strength through heterogeneity of bundled fibrils. J. R. Soc. Interface.

[21] Lin N., Liu X.Y. (2015). Correlation between hierarchical structure of crystal networks and macroscopic performance of mesoscopic soft materials and engineering principles. Chem. Soc. Rev..

[22] Gerberich W.W., Michler J., Mook W.M., Ghisleni R., Östlund F., Stauffer D.D., Ballarini R. (2009). Scale effects for strength, ductility, and toughness in “brittle” materials. J. Mater. Res..

[23] Sui X.M., Tiwari M., Greenfeld I., Khalfin R.L., Meeuw H., Fiedler B., Wagner H.D. (2019). Extreme scale-dependent tensile properties of epoxy fibers. Express Polym. Lett..

[24] Skelton R., de Oliveira M. (2010). Tensegrity Systems.

[25] Van Beek J.D., Hess S., Vollrath F., Meier B.H. (2002). The molecular structure of spider dragline silk: Folding and orientation of the protein backbone. Proc. Natl. Acad. Sci. USA.

[26] Thiel B.L., Guess K.B., Viney C. (1997). Non-periodic lattice crystals in the hierarchical microstructure of spider (major ampullate) silk. Biopolym. Orig. Res. Biomol..

[27] Simmons A.H., Michal C.A., Jelinski L.W. (1996). Molecular orientation and two-component nature of the crystalline fraction of spider dragline silk. Science.

[28] Fornes R.E., Work R.W., Morosoff N. (1983). Molecular orientation of spider silks in the natural and supercontracted states. J. Polym. Sci..

[29] Madurga R., Plaza G.R., Blackledge T.A., Guinea G.V., Elices M., Pérez-Rigueiro J. (2016). Material properties of evolutionary diverse spider silks described by variation in a single structural parameter. Sci. Rep..

[30] Termonia Y. (1994). Molecular modeling of spider silk elasticity. Macromolecules.

[31] Timoshenko S.P., Gere J.M. (1961). Theory of Elastic Stability.

[32] Pérez-Rigueiro J., Madurga R., Gañán-Calvo A.M., Elices M., Guinea G.V., Tasei Y., Asakura T. (2019). Emergence of supercontraction in regenerated silkworm (bombyx mori) silk fibers. Sci. Rep..

[33] Liu X., Zhang K.-Q., Lesieur C. (2014). Oligomerization of Chemical and Biological Compounds, Chapter: Silk Fiber—Molecular Formation Mechanism, Structure—Property Relationship and Advanced Applications.

[34] Patil S.P., Markert B., Gräter F. (2014). Rate-dependent behavior of the amorphous phase of spider dragline silk. Biophys. J..

[35] Keten S., Xu Z., Ihle B., Buehler M.J. (2010). Nanoconfinement controls stiffness, strength and mechanical toughness of β-sheet crystals in silk. Nat. Mater..

[36] Poza P., Pérez-Rigueiro J., Elices M., Llorca J. (2002). Fractographic analysis of silkworm and spider silk. Eng. Fract. Mech..

[37] Brown C.P., MacLeod J., Amenitsch H., Cacho-Nerin F., Gill H.S., Price A.J., Traversa E., Licoccia S., Rosei F. (2011). The critical role of water in spider silk and its consequence for protein mechanics. Nanoscale.

[38] Wan Q., Abrams K.J., Masters R.C., Talari A.C.S., Rehman I.U., Claeyssens F., Holland C., Rodenburg C. (2017). Mapping nanostructural variations in silk by secondary electron hyperspectral imaging. Adv. Mater..

[39] Prez-Rigueiro J., Elices M., Llorca C.V. (2001). Tensile properties of Argiope trifasciata drag line silk obtained from the spider’s web. J. Appl. Polym. Sci..

[40] Garrido M.A., Elices M., Viney C., Pérez-Rigueiro J. (2002). The variability and interdependence of spider drag line tensile properties. Polymer.

[41] Mascolo I., Amendola A., Zuccaro G., Feo L., Fraternali F. (2018). On the geometrically nonlinear elastic response of class θ= 1 tensegrity prisms. Front. Mater..

[42] Grubb D.T., Jelinski L.W. (1997). Fiber morphology of spider silk:  the effects of tensile deformation. Macromolecules.

[43] Liu J., Zhang Y. (2018). Soft network materials with isotropic negative Poisson’s ratios over large strains. Soft Matter.

[44] Pérez-Rigueiro J., Elices M., Plaza G.R., Real J.I., Guinea G.V. (2006). The influence of anaesthesia on the tensile properties of spider silk. J. Exp. Biol..

[45] Yazawa K., Malay A.D., Masunaga H., Norma-Rashid Y., Numata K. (2020). Simultaneous effect of strain rate and humidity on the structure and mechanical behavior of spider silk. Commun. Mater..

[46] Valentini L., Bittolo Bon S., Tripathi M., Dalton A., Pugno N.M. (2019). Regenerated silk and carbon nanotubes dough as masterbatch for high content filled nanocomposites. Front. Mater. Sci..

[47] Frydrych M., Greenhalgh A., Vollrath F. (2019). Artificial spinning of natural silk threads. Sci. Rep..

[48] Liao X., Dulle M., de Souza E Silva J.M., Wehrspohn R.B., Agarwal S., Forster S., Hou H., Smith P., Greiner A. (2019). High strength in combination with high toughness in robust and sustainable polymeric materials. Science.

[49] Koeppel A., Holland C. (2017). Progress and Trends in Artificial Silk Spinning: A Systematic Review. ACS Biomater. Sci. Eng..

[50] Schaefer C., Laity P.R., Holland C., McLeish T.C.B. (2020). Silk Protein Solution: A Natural Example of Sticky Reptation. Macromolecules.

[51] Koeppel A., Laity P.R., Holland C. (2018). Extensional flow behaviour and spinnability of native silk. Soft Matter.

[52] Dionne J., Lefèvre T., Bilodeau P., Lamarre M., Auger M. (2017). A quantitative analysis of the supercontraction-induced molecular disorientation of major ampullate spider silk. Phys. Chem. Chem. Phys..

[53] Guan J., Vollrath F., Porter D. (2011). Two mechanisms for supercontraction in Nephila spider dragline silk. Biomacromolecules.

