# Supplementary material for: Tensegrity Modelling and the High Toughness of Spider Dragline Silk
Source: Nanomaterials (Basel). 2020 Jul 31;10(8):1510. doi: 10.3390/nano10081510 (PMC7466511; doi:10.3390/nano10081510)
Supplement: Supplementary file 1 [file nanomaterials-10-01510-s001.zip › nanomaterials-859275-sup.pdf]

nanomaterials

MDPI

Supporting Information

Tensegrity Modelling and the High Toughness of Spider Dragline Silk

Fernando Fraternali [1,*], Nicola Stehling [2], Ada Amendola [1], Bryan Andres Tiban Anrango [3], Chris Holland [2], and Cornelia Rodenburg [2]

[1] Department of Civil Engineering, University of Salerno, 84084 Fisciano (SA), Italy; adamendola1@unisa.it
[2] Department of Materials Science & Engineering, University of Sheffield, Sir Robert Hadfield Building, Mappin Street, Sheffield, S1 3JD, UK; nastehling1@sheffield.ac.uk (N.S.), christopher.holland@sheffield.ac.uk (C.H.), c.rodenburg@sheffield.ac.uk (C.R.)
[3] Centre for Biomedical and Chemical Science School of Science, Auckland University of Technology, 1010 Auckland, New Zealand; andres.tiban.anrango@aut.ac.nz
* Correspondence: f.fraternali@unisa.it

1. Collection and Understanding Low Voltage SEM Images

For SEM imaging, the silk fibres were attached to an aluminium stub and joined to the stub edges using adhesive tape. Conductive silver paint was applied to the extremes of the fibre to form a connection to ground for SEM imaging. The lens detector in an FEI Nova NanoSEM 450 was used to collect secondary electron images with the following parameters: accelerating voltage of 1 kV, spot size of 3, and a working distance of 3 mm. Images were acquired using a 50 ns dwell time, scan interlace of 8, and an image integration of 32.

For interpretation of the low voltage SEM, its higher resolution and surface sensitivity, compared to standard SEM, need to be considered for the interpretation of nanostructures, as demonstrated previously [1]. A consequence of the high surface sensitivity, when using primary beam voltages of 1kV as in our experiments, is that the assembly of microcrystalline granules into nanofibrils can only be observed in areas were the nanofibril is exactly parallel to the surface. This is the case at the bottom of Figure S1. Due to the curvature of the fibre, the topography becomes more clearly visible near the fibre edge (bottom of Figure S1) as well, revealing that fibrillar patters exist. That these consist of individual granules is seen better in the insert where the contrast was enhanced. Small misalignments of the nanofibrils with respect to the fibre surface in conjunction with the high surface sensitivity of low voltage SEM is responsible for discontinuous appearance in much of the remainder of the fibre. Misorientation varies for different layers: longer chains become visible away from the fibre edge in Figure 1c of the main manuscript. Note that nanofibrils can also be observed in LV-SEM, when the skin layer was removed chemically, followed by an enzymatic treatment [2].

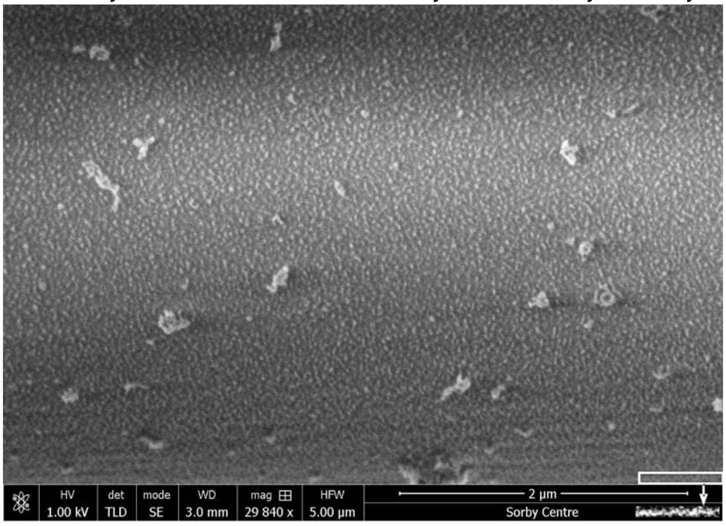

Figure S1. SEM image of the edge of the fibre after air plasma exposure to 66W for 1.5 min.

2. Optimisation of Plasma Exposure Times and Layer Identification

Optimising the plasma treatment required balancing of the overall exposure time with the ability to resolve at which fibre diameter significant nanostructure changes occur. Lower plasma power will result in a higher depth resolution, as less material is removed in each step, but requires a much larger number of exposures to reach the same depth. Therefore, a plasma power of 66W was chosen (reduced from 100W in [3]). The plasma etching was carried out in Diener Electronic Zepto plasma cleaner; all other parameters were kept the same as in [3] e.g., 40 kHz, 100 W and 0.3 mbar pressure with lab air as the process gas. The power level of 66W was based on time series experiments carried out on one fibre to establish the exposure times that reveal key transitions in the nanostructure as shown below.

Figure S2 shows the fibre prior to any plasma exposure on the left, which does not exhibit any nanostructures.

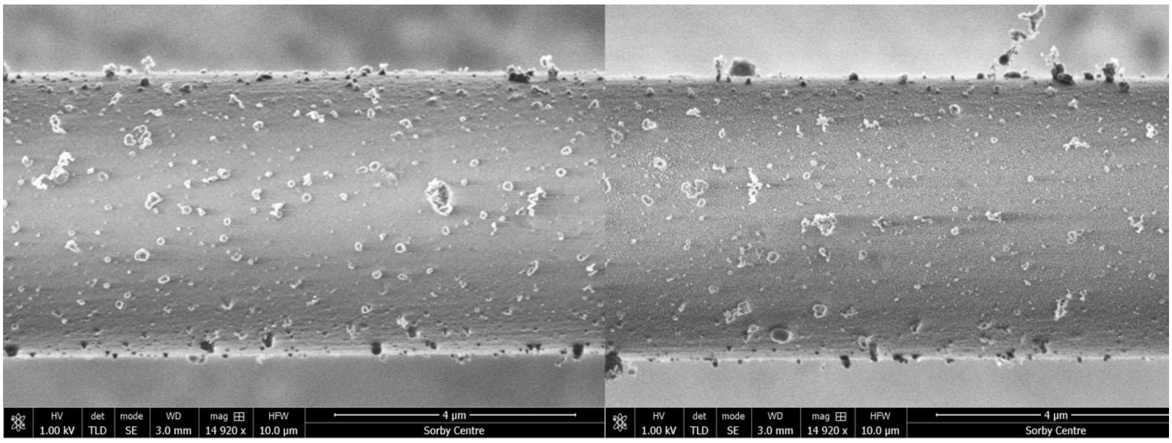

Figure S2. left: untreated fibre, **right**: after 1 min exposure to plasma.

After 1 min plasma exposure, a faint indication of network becomes visible in some areas, akin to that when the skin layer was removed by chemical treatment. After 2 min plasma exposure (see Figure S3, left), these nanostructures were clearly visible throughout the fibre; thus, 1.5 min plasma treatment was chosen in order to capture the outer edge of layer 1 in the main manuscript.

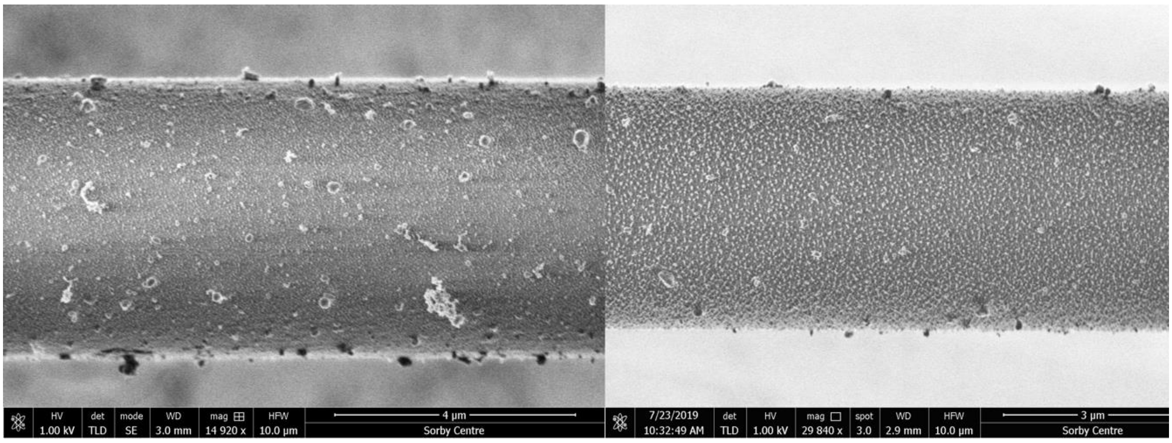

Figure S3. left: after 2 min exposure to plasma, **right**: after 6 min exposure to plasma.

The same fibre was used in further exposure steps (1 min steps). At 6 min (Figure S3, right), another notable transition takes place. The nanostructures are larger and longer chains of granules are visible notably; moreover, these are in angles to the fibre axis. The uniform reduction in fibre diameter with etch time is also evident by comparing Figures S2 and S3. That the reductions in

diameter are consistent with a layered structure was already demonstrated in [3]. While it is possible to prepare cross-sections with considerable difficulty [4], imaging in an edge on orientation using magnifications high enough to resolve the nanostructures is hampered by charging, as can be seen in Figure 6.1f in [4]. Thus, the plasma etching technique was developed to provide the quantitative inputs required for our model.

To collect the data in Figure 1 in the main manuscript, a different fibre was used, and it was only exposed to the treatment times deemed critical from the above time series experiment. In order to avoid any temperature increase as a result of a prolonged continuous exposure, the treatment was carried out in intervals of 2 min plasma exposure, with 2 min resting time between exposures.

References

[1] Rodenburg, C.; Rainforth. W.M. The influence of beam energy and oxidation on quantitative carbide analysis in the scanning electron microscope. *J. Appl. Phys.* **2006,** *100*, 114902.

[2] Sogawa, H., Nakano, K., Tateishi, A., Tajima, K., & Numata, K. Surface analysis of native spider draglines by FE-SEM and XPS. *Front. Bioeng. Biotechnol.* **2020**, *8*, 231.

[3] Stehling, N.; Abrams, K.J.; Holland, C.; Rodenburg, C. Revealing Spider Silk's 3D Nanostructure Through Low Temperature Plasma Etching and Advanced Low-Voltage SEM. *Front. Mater.* **2019**, *5*, 84.

[4] Stehling, N.A. Scanning Electron Microscopy for Nano-morphology Characterisation of Complex Hierarchical Polymer Structures. Ph.D. Thesis, University of Sheffield, 2019.
